# P-977. Design and Implementation of a State-Wide Infection Prevention and Control (IPC) Curriculum; Delivering Comprehensive, Engaging, Accessible Content Promotes Sustained Learner Engagement

**DOI:** 10.1093/ofid/ofae631.1167

**Published:** 2025-01-29

**Authors:** Patrick R Ching, Barry Rittmann, Kaila Cooper, Jo Dee Armstrong-Novak, Michael Stevens, Angela Spleen, Sarah Lineberger, Ginger Vanhoozer, Rachel Pryor, Sangeeta Sastry, Ryan Wooten, Michelle Doll

**Affiliations:** Virginia Commonwealth University School of Medicine, Richmond, Virginia; Virginia Commonwealth University Health System, Richmond, Virginia; Virginia Commonwealth University Health System, Richmond, Virginia; Virginia Commonwealth University Health System, Richmond, Virginia; West Virginia University, Morgantown, West Virginia; Virginia Department of Health, Richmond, Virginia; Virginia Department of Health, Richmond, Virginia; Virginia Department of Health, Richmond, Virginia; Virginia Hospital and Healthcare Association, Richmond, Virginia; VCU, Richmond, Virginia; Virginia Commonwealth University Health System, Richmond, Virginia; Virginia Commonwealth University Health System, Richmond, Virginia

## Abstract

**Background:**

In the wake of COVID-19, the Virginia Department of Health (VDH) partnered with Virginia Commonwealth University Health System to produce a comprehensive IPC curriculum to address known gaps in education among healthcare providers. A 2021 needs assessment conducted by VDH found needs for comprehensive IPC education that was more accessible, engaging, and interactive.

Core Content Attendee Work Settings

**Figure:**
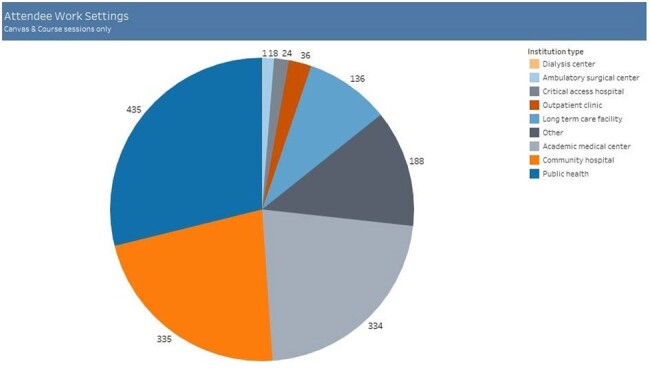

**Methods:**

A tiered curriculum was developed to address the identified needs from a variety of healthcare provider types: Foundational, Intermediate, and Advanced, targeting respectively concepts critical for anyone in the healthcare environment, concepts for HCP actively engaged in IPC activities in their facility, and concepts for those leading IPC programs or healthcare facilities. The education was delivered in a hybrid model with virtual-synchronous and virtual-asynchronous offerings in addition to in-person conferences. To rapidly build engaging content, expertise to create content was contracted from experts in IP across the United States. Evaluations and content created from 7/2021-4/2024 were reviewed and summarized.

Total Attendees by Learning Experience

**Figure:**
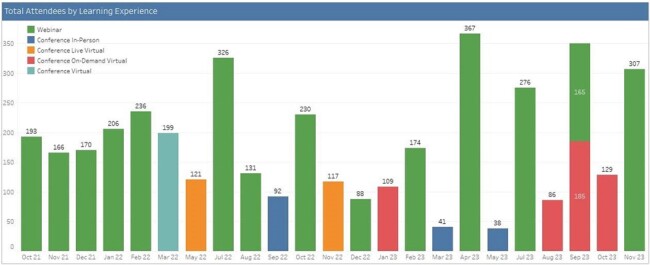

**Results:**

Eighty individual modules organized into 3 core courses, a webinar series, and specialty site series were created by 62 individual IPC experts in less than 3 years. The program conducted 4 in-person conferences and 2 additional synchronous virtual conferences, and 13 live webinars. Materials remain accessible for on-demand learning. Content has been consumed by 4152 attendees (by total # registrations, not unique participants) across 43 states and 19 different countries. The breakdown of learner types is shown in Figure 1. Learners were more likely to access live virtual content (3472) than on-demand virtual content (509) or in-person events (171). Total attendees by event type over time are shown in Figure 2.

**Conclusion:**

By leveraging existing expertise within the Infection Prevention community of professionals, a comprehensive IP program was rapidly implemented and provided sustained educational opportunities to HCP. HCPs preferred live virtual events to other delivery models to access the content.

**Disclosures:**

**All Authors**: No reported disclosures

